# Radiation-resistant cancer stem-like cell properties are regulated by PTEN through the activity of nuclear β-catenin in nasopharyngeal carcinoma

**DOI:** 10.18632/oncotarget.20339

**Published:** 2017-08-18

**Authors:** Gong Zhang, Wenjun Wang, Chunxiao Yao, Shuping Zhang, Lili Liang, Muyuan Han, Jinjin Ren, Xiurong Qi, Xiaofeng Zhang, Shuye Wang, Lei Li

**Affiliations:** ^1^ Department of Radiotherapy of People’s Hospital of Shanxi Province, Taiyuan 030012, PR China; ^2^ Research Institute of Respiratory Diseases, The First Affiliated Hospital of Guangzhou Medical University, Guangzhou 510120, PR China; ^3^ Department of Dermatology of People’s Hospital of Shanxi Province, Taiyuan 030012, PR China; ^4^ Department of Ophthalmology of People’s Hospital of Shanxi Province, Taiyuan 030012, PR China

**Keywords:** PTEN, nuclear β-catenin, cancer stem-like cells, radioresistance, nasopharyngeal carcinoma

## Abstract

Radiotherapy is the primary and most important treatment for nasopharyngeal carcinoma (NPC). Cancer stem-like cells (CSCs) have been shown to be resistant to radiation. The phosphatase and tensin homolog deleted on chromosome 10 (PTEN) tumor suppressor gene has been suggested to play a role in stem cell self-renewal. In the present study, we sorted PTEN−/+ cells using a flow cytometer. The clone formation assay showed that PTEN− cells were more radioresistant than PTEN+ NPC cells. We found that PTEN− cells demonstrated a significant increase in tumorsphere formation and CSCs markers compared with PTEN+ cells. Silencing the expression of PTEN with siRNA resulted in increased expression of p-AKT, active β-catenin and Nanog. siPTEN cells irradiated showed more radioresistant and DNA damage than parental cells. We also confirmed that down-regulation of β-catenin expression with shRNA resulted in a reduced percentage of side population cells and expression of Nanog. shβ-catenin cells significantly decreased survivin expression at 4 Gy irradiation in PTEN− cells compared with PTEN+ cells. In siPTEN cells, β-catenin staining shifted from the cytoplasmic membrane to the nucleus. Furthermore, immunofluorescence showed that following irradiation of PTEN− cells, at 4 Gy, active β-catenin was mainly found in the nucleus. Immunohistochemistry analysis also demonstrated that the PTEN−/p-AKT+/β-catenin+/Nanog+ axis may indicate poor prognosis and radioresistance in clinical NPC specimens. Thus, our findings strongly suggest that PTEN− cells have CSCs properties that are resistant to radiation in NPC. PTEN exerts these effects through the downstream effector PI3K/AKT/β-catenin/Nanog axis which depends on nuclear β-catenin accumulation.

## INTRODUCTION

Radiotherapy is the primary and most important treatment modality for NPC [1]. The loco-regional control rate of NPC has significantly improved in the past decade. Even though there have been significant improvements in the loco-regional control rates of NPC, local recurrence still contributes to concerning levels of mortality and morbidity. The management of local failure in advanced stages of NPC still presents a difficult challenge [2]. Radiobiological research over the past decades has provided evidence that the number of cancer stem-like cells (CSCs) and the intrinsic radiosensitivity of CSCs affect radiocurability [3]. Notably, recurrence and metastasis may arise from residual disease resulting from the ability of CSCs to resistant radiation, which has been demonstrated both experimentally and clinically [4].

It has been suggested that the phosphatase and tensin homolog deleted on chromosome 10 (PTEN) tumor suppressor gene, one of the most commonly mutated genes in human carcinomas, plays a role in stem cell self-renewal [5]. PTEN can regulate side population (SP) cells which have been identified as stem-like cells [6]. In addition, PTEN has been found as a prognostic marker in postoperative radiotherapy for cancer of the head and neck [7]. PTEN functions as a phosphatase by catalyzing the dephosphorylation of the 3-phosphate of the inositol ring in phosphatidylinositol 3,4,5-trisphosphate (PIP3), resulting in the biphosphate product phosphatidylinositol 4,5-bisphosphate. PIP3 is critical in the activation of AKT, therefore its dephosphorylation results in the inactivation of the PI3K/AKT signal pathway [8]. It was reported that the PTEN/PI3K/AKT pathway regulates stem-like cells in some primary carcinomas [9–11]. It is known that the PI3K/AKT signaling pathway has been correlated with radioresistance [12–13]. However, the PI3K/AKT signaling pathway has not been fully elucidated even though its activation is correlated with radioresistance. In the present study, we detected the possible molecular mechanism underlying CSCs and their radioresistance in NPC.

Several signaling pathways have been found to be involved in CSCs regeneration. [14]. A large number of cancers have been implicated in the maintenance of CSCs via modulation of the Wnt/β-catenin signaling axis [15]. The main purpose of the Wnt pathway is the stabilization and translocation of β-catenin into the nucleus. In the nucleus, β-catenin increases the expression of several genes targeted by the Wnt pathway by binding to the TCF/LEF family of transcription factors [16]. Nanog is reported to be a target gene of β-catenin and is thought to be one of four factors that reprograms adult cells into induced pluripotent stem (iPS) cells.[17]. Nanog is critically involved in the regulation of CSCs in several types of tumor [18]. Differentiation is inhibited when the expression of Nanog is upregulated by β-catenin [19].

In the present study, we investigated the role of PTEN in the self-renewal and radioresistance of NPC CSCs. We found that inhibition of PTEN increased the number of CSCs and their radioresistance. The effect of PTEN was mediated by PI3K/AKT/β-catenin/Nanog signaling. We also found that nuclear β-catenin activation is essential for regulating the CSCs phenotype and radioresistance of NPC cells. Thus, our findings reveal that the PTEN/PI3K/AKT/β-catenin/Nanog axis is a potential signaling pathway for CSCs and radioresistance of NPC, which may have potential clinical implications for the treatment of NPC.

## RESULTS

### PTEN− cells are capable of self-renewal and are resistant to doses of radiation

**Figure 1:**
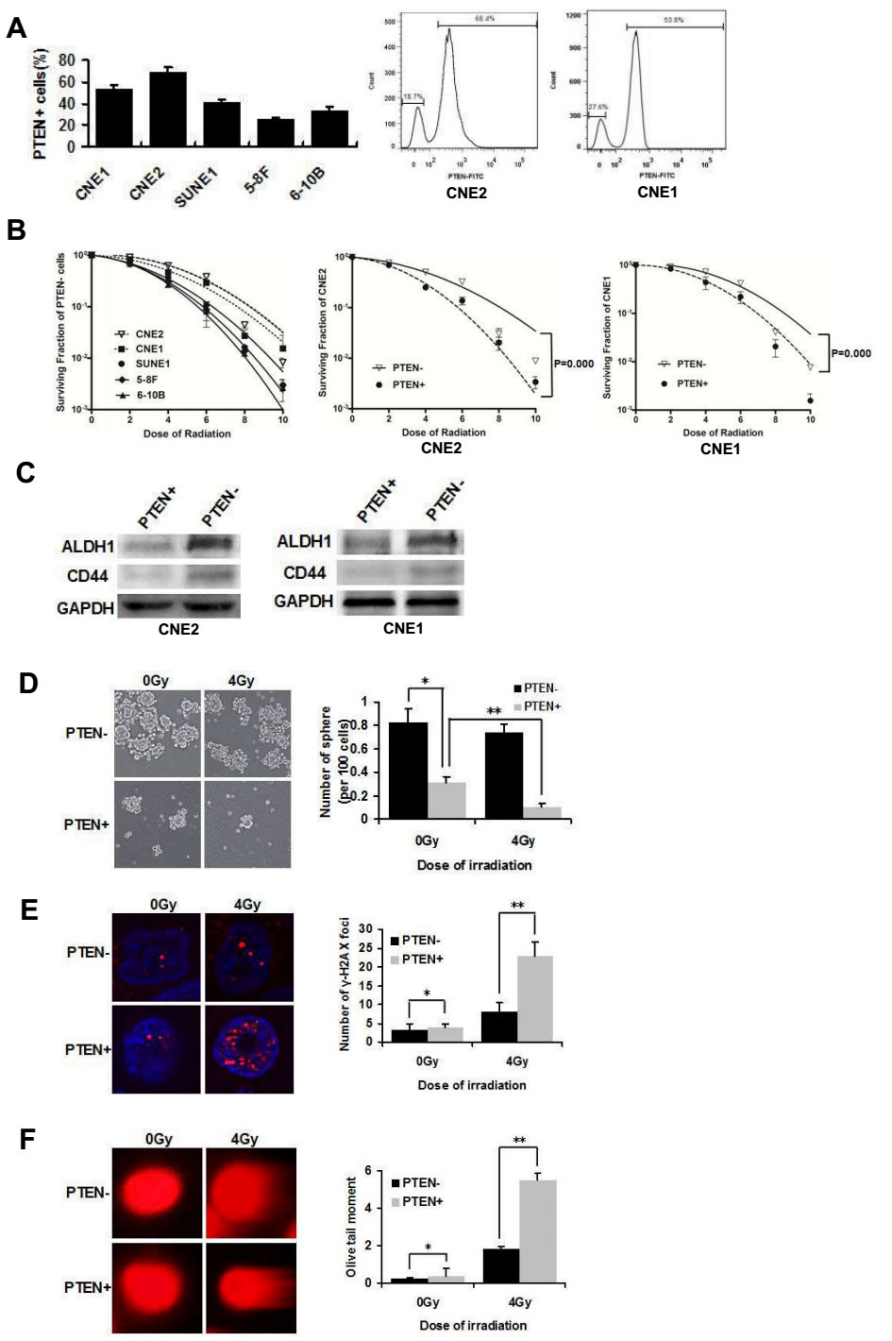
PTEN− cells are capable of self-renewal and are resistant to radiation **(A)** Expression levels of PTEN+ cells in five NPC cell lines. CNE2 and CNE1 cells were stained with PTEN antibody, and analyzed by flow cytometry. Two populations can be distinguished based on PTEN fluorescence: PTEN−/CNE2 18.7% and PTEN+/CNE2 68.4%, PTEN−/CNE1 36.4% and PTEN+/CNE1 53.8%. **(B)** PTEN- cells in five NPC cell lines CNE2, CNE1, SUNE1, 5-8F and 6-10B were irradiated with 0, 2, 4, 6, 8 or 10 Gy and a colony formation assay was conducted (Left). Survival curves of PTEN−/+ CNE2 and PTEN−/+ CNE1 cells (middle and right). **(C)** Western blot analysis of the expression of ALDH1 and CD44 in PTEN−/+ CNE2 and CNE1 cells. **(D)** Tumorsphere formation in PTEN− and PTEN+ irradiated CNE2 cells cultivated in serum-free medium for 72 h. * and ** indicate P < 0.01. **(E)** PTEN− and PTEN+ CNE2 cells were sorted directly onto glass slides following irradiation at 0Gy or 4 Gy and immunostained with anti-γ-H2AX (red). Nuclei were stained with DAPI (blue). There were significantly more DNA-damaged foci in the PTEN+ population. * indicates P > 0.05, ** indicates P < 0.01. **(F)** Detection of DNA damage of PTEN− and PTEN+ irradiated CNE2 cells by comet assay. * indicates P > 0.05, ** indicates P < 0.01. Data are shown as mean ± S.D of three independent experiments.

In our previous research, fluorescent-activated cell sorting (FACS) analyses revealed that CNE2 and CNE1 cells had higher levels of PTEN than other cells in five NPC cell lines (CNE1, CNE2, SUNE1, 5-8F, 6-10B). Figure 1A showed that PTEN+ cells accounted for 68.4% and 53.8% in CNE2 and CNE1 cells, respectively. Importantly, the clone formation assay showed that PTEN− cells of CNE2 and CNE1 cells were more radioresistant than the other three cell lines (Figure 1B, CNE2> CNE1> SUNE1> 5-8F> 6-10B). Thus, the CNE2 and CNE1 cell lines were chosen for this study and were poorly differentiated NPC cell lines. CNE2 and CNE1 cells were stained with primary antibody against PTEN and sorted into PTEN−/+ cells. The clone formation assay showed that PTEN− cells of CNE2 and CNE1 cells were more radioresistant than PTEN+ cells (Figure 1B).

Westernblot analysis of representative NPC stem cell markers, ALDH1 and CD44 [20], revealed increased expression in PTEN−/CNE2 and PTEN−/CNE1 cells, relative to PTEN+ cells (Figure 1C). As a functional measure of CSCs frequency [21], we tested the tumorsphere forming ability of PTEN−/+ CNE2 cells. Cells were irradiated at 0 Gy (sham irradiation) and 4 Gy. PTEN− and PTEN+ populations were sorted by FACS, and then the cells were cultivated in suspension. Floating tumor spheroids were observed after 72 h of culture. PTEN− cells demonstrated a significant increase in tumorsphere formation compared to PTEN+ cells. There was no significant difference in tumorsphere forming ability before and after radiation of PTEN− cells. However, after radiation of PTEN+ cells, tumor spheroid formation was significantly inhibited (Figure 1C).

To identify differences in radiation-induced DNA damage between PTEN− and PTEN+ cells, we examined foci of DNA damage using γ-histone 2AX (γ-H2AX) as a DNA-damage marker in CNE2 cells with confocal microscopy [22]. Immunostaining of γ-H2AX following irradiation at 4 Gy exhibited that PTEN+ cells contained more γ-H2AX foci than PTEN− cells (Figure 1E). The alkaline comet assay mainly measures strand breaks which are important in irradiation-induced lethality [23]. As with the comet assay, we observed 4Gy irradiation caused damage to DNA in PTEN+/CNE2 cells were more than PTEN− cells by analyzing the olive tail moment (Figure 1F). These results suggested that PTEN− cells have CSCs properties which are resistant to radiation in NPC.

### PTEN regulates CSCs phenotypes and radioresistance through the PI3K/AKT/β-catenin pathway

**Figure 2:**
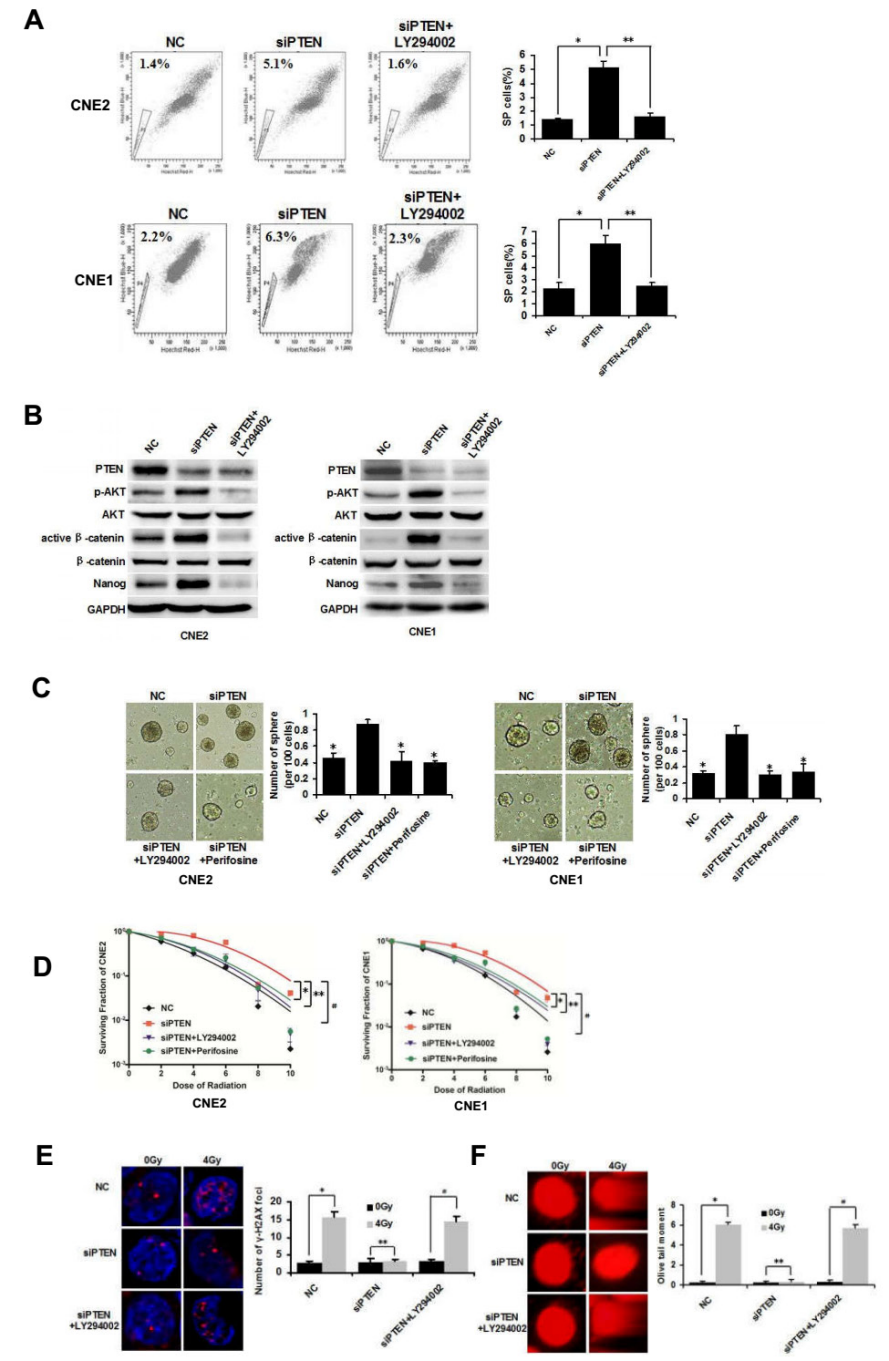
PTEN regulates CSCs and radioresistance through the PI3K/AKT/β-catenin pathway **(A)** CNE2 and CNE1 cells were transfected with NC or siRNA targeting PTEN. FACS analysis of SP cells in NC, siPTEN and siPTEN+LY294002 CNE2 and CNE1 cells. * and ** indicate P < 0.01, vs. siPTEN group. **(B)** Western blot analysis of the expression of PTEN, AKT, p-AKT, β-catenin, active β-catenin and Nanog in NC, siPTEN and siPTEN+LY294002 CNE2 and CNE1 cells. **(C)** Tumorsphere formation in NC, siPTEN, siPTEN+LY294002 and siPTEN+Perifosine CNE2 and CNE1 cells cultivated in serum-free medium for 72 h. * indicates P < 0.01, vs. siPTEN group. **(D)** Survival curves of NC, siPTEN, siPTEN+LY294002 and siPTEN+Perifosine CNE2 and CNE1 cells.*, ** and # indicate P < 0.01, vs. siPTEN group. **(E)** NC, siPTEN and siPTEN+LY294002 CNE2 cells were sorted directly onto glass slides following irradiation at 0Gy or 4 Gy and immunostained with anti-γ-H2AX (red). Nuclei were stained with DAPI (blue). * and # indicate P < 0.01, ** indicates P > 0.05. **(F)** Detection of DNA damage of NC, siPTEN and siPTEN+LY294002 CNE2 cells by comet assay. * and # indicate P < 0.01, ** indicates P > 0.05. Data are shown as mean ± S.D of three independent experiments.

PTEN is known to inhibit the PI3K/AKT signaling axis, and β-catenin is known to mediate the effect of AKT signaling on stem cells [11]. We assessed whether this pathway mediated the regulatory function of NPC CSCs phenotypes. To this end, PTEN was knocked down with siRNA in CNE2 and CNE1 cells. Down-regulation of PTEN increased the percentage of SP cells. LY294002 is a commonly used PI3K inhibitor. Inhibition of PI3K by LY294002 (15 μM) reversed the increase in SP cells in siPTEN/CNE2 and siPTEN/CNE1 cells (Figure 2A). Moreover, the effects of downstream effectors were examined using western blot. As shown in Figure 2B, compared to NC cells, siPTEN/CNE2 and siPTEN/CNE1 cells have marked increases in the phosphorylation levels of AKT, non-phosphorylated β-catenin (active β-catenin) and Nanog, indicating activation of the PI3K/AKT/β-catenin pathway and its downstream CSCs related protein Nanog. LY294002 (15 μM) treatment reversed the increase in downstream proteins in siPTEN cells (Figure 2B).

To further characterize AKT in PI3K/AKT/β-catenin pathway, we treated the siPTEN cells with Akt inhibitor Perifosine (2 μM) [24]. As demonstrated in Figure 2C, siPTEN/CNE2 and siPTEN/CNE1 cells showed increased tumorsphere formation, compared to control NC cells. However, PI3K and AKT inhibitor suppressed the formation of tumorspheres in siPTEN cells obviously. The clone formation assay showed that siPTEN/CNE2 and siPTEN/CNE1 cells were more radioresistant than NC control cells. LY294004 and Perifosine treatment significantly decreased the radioresistance of siPTEN cells (Figure 2D).

Immunostaining demonstrated that siPTEN/CNE2 cells contained more γ-H2AX foci than the control NC/CNE2 cells following irradiation at 4 Gy. siPTEN/CNE2 cells exhibited no obvious further increase in γ- H2AX foci induced by 4 Gy irradiation. LY292004 treatment can obviously increase γ-H2AX foci in siPTEN cells (Figure 2E). As with the comet assay, we observed 4 Gy irradiation caused little damage to DNA in siPTEN/CNE2 cells. LY294002 treatment caused obvious damage to DNA in siPTEN/CNE2 cells (Figure 2F). To sum up, these findings provide mechanistic evidence for the involvement of the PTEN/PI3K/AKT/β-catenin axis in promoting CSCs properties and radioresistance in NPC.

### NPC CSCs radioresistance is regulated through downstream β-catenin signaling

**Figure 3:**
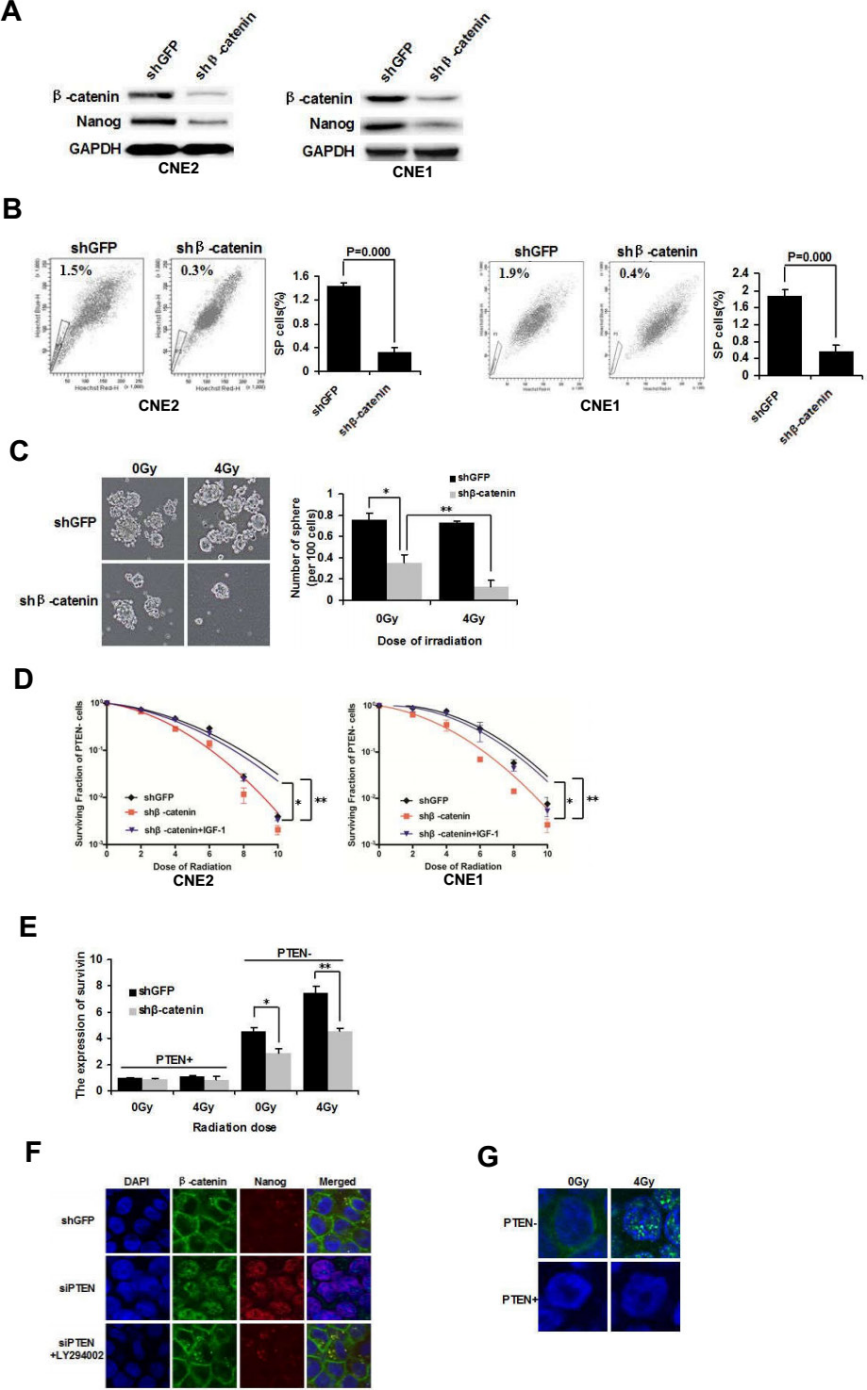
β-catenin nuclear accumulation is involved in mediating NPC CSCs and radioresistance **(A)** β-catenin was knocked down with lentivirus-mediated shRNA in CNE2 and CNE1 cells. Western blot analysis of the expression of β-catenin and Nanog. **(B)** FACS analysis of SP cells in shGFP and shβ-catenin CNE2 and CNE1 cells. **(C)** Tumorsphere formation of irradiated PTEN− cells in shGFP and shβ-catenin CNE2 cells cultivated in serum-free medium for 72 h. **(D)** Survival curves of PTEN- cells in shGFP, shβ-catenin and shβ-catenin+IGF-1 CNE2 and CNE1 cells. **(E)** shGFP and shβ-catenin CNE2 cells were irradiated at 0 Gy and 4 Gy. After 24 h the irradiated cells were sorted into PTEN− and PTEN+ populations. The expression of survivin was determined by qPCR analysis. * and ** indicate P < 0.05. **(F)** In shGFP control CNE2 cells, β-catenin was mainly located in cytoplasmic membranes. Inhibition of β-catenin resulted in increased β-catenin nuclear staining, which was reversed by LY294002 (15 μM). **(G)** Immunofluorescence of active β-catenin in PTEN− and PTEN+ cells after 0 Gy and 4 Gy irradiation. β-catenin is shown in green, and the nuclei are stained with DAPI (blue). Images were captured by confocal microscopy.

In a previous study, we demonstrated that β-catenin is inhibited in siPTEN cells by LY294002. To further examine whether β-catenin is required for NPC CSCs, β-catenin was knocked down by lentivirus-mediated shRNA in CNE2 and CNE1 cells. Compared with the control shGFP cells, CNE2 and CNE1 cells infected with shβ-catenin lentivirus exhibited decreased expression of β-catenin and Nanog (Figure 3A). Furthermore, a decreased percentage of SP cells was also observed in shβ-catenin CNE2 and CNE1 cells (Figure 3B).

Furthermore, shβ-catenin CNE2 cells were irradiated at 0 Gy and 4 Gy. The PTEN− population was sorted by FACS and the cells were then cultivated in suspension. shβ-catenin PTEN− cells demonstrated a significant decrease in tumorsphere formation compared with shGFP PTEN− cells at 0 Gy and 4 Gy irradiation. In addition, following irradiation of shβ-catenin PTEN− cells, tumorsphere formation ability was significantly inhibited at 4 Gy irradiation, compared to sham irradiation (Figure 3C). The clone formation assay showed that PTEN− cells of shβ-catenin CNE2 and CNE1 cells were more radiosensitive than PTEN− shGFP cells. Insulin-like growth factor-1 (IGF-1, 100 ng/mL), a PI3K/AKT activator [25] treatment for 72 h reversed the increased radiosensitivity of PTEN− cells in shβ-catenin CNE2 and CNE1 cells (Figure 3D). The result indicated that PTEN/PI3K/AKT/β-catenin axis can revert the radio-resistance characteristic of PTEN− cells.

Survivin, a bifunctional member of the apoptosis family, is another direct target of β-catenin [26]. Survivin plays an important role in radioresistance [27]. To determine whether survivin is differentially mediated in response to radiation, shGFP and shβ-catenin CNE2 cells were irradiated at 0 or 4 Gy. Cells were harvested after 24 h and then PTEN− and PTEN+ populations were sorted by FACS. Radiation selectively up-regulated survivin expression in PTEN− cells at 4 Gy (shGFP PTEN−, 0 Gy vs. 4 Gy, P< 0.05; Figure 3E) as shown by qPCR analysis. As β-catenin directly activates survivin, inhibition of β-catenin significantly decreases survivin expression at 0 Gy and 4 Gy (PTEN−, sh-GFP vs. sh-β-catenin P< 0.05; Figure 3E). Accordingly, the survivin level is elevated further following irradiation of sh-β-catenin transduced PTEN− cells (shβ-catenin PTEN−, 0 Gy vs. 4 Gy P< 0.05; Figure 3E). However, radiation did not enhance survivin level in PTEN+ cells. In addition, transduction with shβ-catenin did not down-regulated the basal level of survivin in PTEN+ cells. These studies indicate that β-catenin plays a key role in the radioresistance of NPC, preferentially in PTEN− stem-like cells.

### Nuclear β-catenin localization is essential for maintaining stemness and radioresistance

Nuclear β-catenin accumulation is essential for transcriptional activity of β-catenin, which can regulate endonuclear stem cell related transcription factors, such as Nanog [28]. The activity of nuclear β-catenin depends on PI3K signaling [29]. The effect of PTEN/PI3K/AKT signaling on the subcellular localization of β-catenin in CNE2 cells was examined by immunofluorescence. β-catenin was located predominantly at the plasma membrane in shGFP CNE2 control cells, with only faint signals observed in the cytoplasm. Nanog was not activated. When PTEN was down-regulated, β-catenin staining transferred from the cytoplasmic membrane to the nucleus. In addition, downstream Nanog expression was observed in the nucleus of siPTEN/CNE2 cells. When siPTEN/CNE2 cells were treated with LY294002 (15 μM), PI3K/AKT signaling was abolished, β-catenin was still expressed in the plasma membrane and Nanog was not activated (Figure 3F). These results showed that the transcriptional activity of nuclear β-catenin depends on PTEN/PI3K/AKT axis activity. β-catenin can up-regulate Nanog expression which may result in CSCs properties.

After establishing that PTEN− cells have CSCs properties and we next determined whether nuclear β-catenin plays a role in the radioresistance of PTEN− cells. Confocal microscopy indicated that active β-catenin was mainly localized around the cell membrane in PTEN−/CNE2 cells, whereas it was difficult to examine β-catenin in PTEN+ cells. Following irradiation of PTEN−/CNE2 cells at 4 Gy, active β-catenin was located mainly in the nucleus rather than around the cell membrane (Figure 3G). PTEN− and PTEN+ cells were found to be intrinsically different with respect to the level of active β-catenin. Interestingly, in PTEN− cells, the localization of β-catenin changed markedly following irradiation. Thus, our results showed that nuclear β-catenin activation is essential for regulating the CSCs phenotype and radioresistance of NPC cells.

### Expression of PTEN, p-AKT, nuclear β-catenin and Nanog are correlated and can predict the prognosis of radiotherapy in human NPC samples

**Figure 4:**
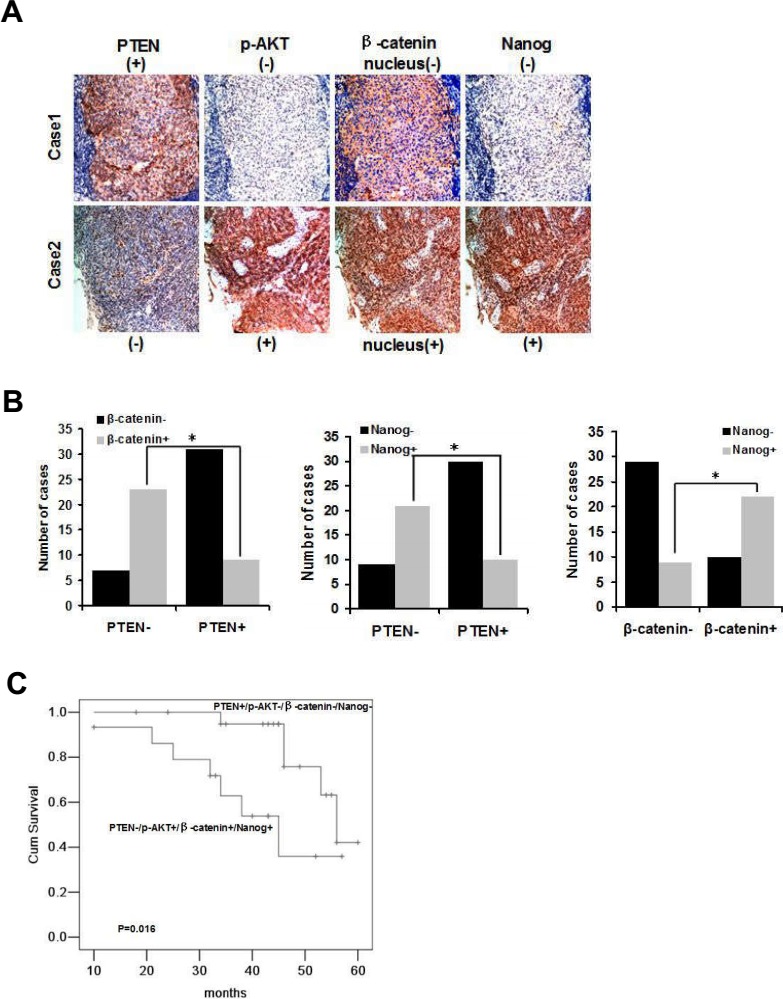
Expression of PTEN, p-AKT, nuclear β-catenin and Nanog is correlated and can predict the prognosis of radiotherapy in NPC samples **(A)** Representative cases from 70 NPC specimens in tissue microarrays were analyzed by immunohistochemical staining (PTEN, p-AKT, nuclear β-catenin and Nanog). Photographs were taken at ×200 magnification. **(B)** Graphs summarizing chi-squared analysis of immunohistochemical staining of PTEN vs. β-catenin, * indicates P = 0.000; PTEN vs. Nanog, * indicates P = 0.000; β-catenin vs. Nanog, * indicates P = 0.000. **(C)** Kaplan-Meier analysis of overall survival of NPC patients who received radiotherapy according to the expression of PTEN−/p-AKT+/nucleus β-catenin+/Nanog+ (n= 14) and PTEN+/p-AKT−/nucleus β-catenin−/Nanog− (n= 21), P= 0.016.

Our experiments demonstrate that CSCs and radioresistance are regulated by PTEN/PI3K/AKT/β-catenin signaling *in vitro*. To determine whether the same effect occurs in primary tumors from NPC patients, we performed an immunohistochemical analysis using antibodies against PTEN, p-AKT, β-catenin, and Nanog on a tumor tissue microarray consisting of paraffin-embedded NPC samples derived from 70 patients (Figure 4A). Immunohistochemical analysis was carried out using continuous paraffin sections. Negative immunostaining for PTEN was found in 30 of 70 tumors (43%). Eighteen of 30 (60%) specimens with negative PTEN staining displayed both nucleus β-catenin and Nanog immunoreactivity. Of the 40 PTEN positive specimens, 25 (63%) showed neither nucleus β-catenin nor Nanog staining. Statistical analysis of these results indicated that PTEN negativity was significantly associated with that of nucleus β-catenin and Nanog (P < 0.01 and P < 0.01, respectively, Figure 4B). Intriguingly, immunoreactivity of nucleus β-catenin also correlated significantly with Nanog (P < 0.01, Figure 4B).

Radiotherapy is the most important treatment for NPC. Of the 70 samples in the present study, 57 patients had received radiotherapy. We analyzed the survival rate of these patients according to the status of PTEN, p-AKT, nucleus β-catenin and Nanog. Patients with PTEN+/p-AKT−/nucleus β-catenin−/Nanog− expression had an obviously better prognosis than patients with PTEN−/p-AKT+/nucleus β-catenin+/Nanog+ expression (P = 0.016, Figure 4C). Thus, the expression of negative PTEN, positive p-AKT, nucleus β-catenin and Nanog were correlated. The PTEN−/p-AKT+/nucleus β-catenin+/Nanog+ axis may indicate poor prognosis and radioresistance of NPC.

## DISCUSSION

CSCs are presumed to drive tumor initiation and tumor radioresistance. NPC recurrence and metastasis often result in treatment failure due to radioresistance [30]. Hence, the poor prognosis of advanced NPC likely results from the failure to target CSCs. PTEN has been identified as a tumor suppressor, which is often deleted or reduced in a variety of cancers and is associated with resistance and relapse in response to conventional therapeutic agents [31–32]. In the present study, we demonstrated that PTEN− cells isolated by FACS induced more tumorspheres as compared to PTEN+ cells. It was surprising to find that the PTEN− subpopulation consisted of progenitor cells. Increased γ-H2AX level and olive tail moment were detected in PTEN+ cells following a single radiation dose. However, radiation did not increase γ-H2AX level and olive tail moment in PTEN− cells. Our data indicate the existence of a more efficient DNA damage checkpoint and repair mechanism in PTEN− stem like cells compared with PTEN+ cells. PTEN has been shown to regulate the DNA damage response pathway. Chk1-mediated checkpoint activation is impaired when PTEN is absent owing to the cytoplasmic sequestration of ubiquitinated Chk1. In response to ionizing radiation a partially defective checkpoint is found in PTEN null cells [33].

In our research, we showed that PTEN inhibition resulted in an increased SP cells and tumorsphere formation. LY294002 treatment attenuated increased SP cells and tumorsphere formation in siPTEN cells. In addition, AKT inhibitor Perifosine inhibited tumosphere formation of siPTEN cells. LY294002 and Perifosine can promoted the radiosensitivity of siPTEN cells. These results suggest that down-regulation of PTEN increased the proportion of CSCs and radioresistance, which was mainly dependent on the PI3K/AKT pathway.

As Wnt signaling mediated by β-catenin has been exhibited to play a role in stem cell self-renewal. The proportion of SP cells and the expression of Nanog were significantly reduced after silencing the expression of β-catenin. shβ-catenin increased radiosensitive to irradiation of PTEN− cells which can be abolished by PI3K/AKT activator IGF-1. These results indicated that β-catenin contributed to the development of CSCs and radioresistance through PTEN/PI3K/AKT/β-catenin axis. Furthermore, the selective decrease in survivin expression level in shβ-catenin PTEN− cells indicates that survivin may be a mechanism regulated partly through the Wnt/β-catenin pathway, which elevates radioresistance in the progenitor PTEN− cells.

Activated AKT leads to the nuclear translocation and activation of β-catenin through phosphorylation and inactivation of GSK3-β, resulting in nuclear translocation and activation of β-catenin. Furthermore, AKT can directly phosphorylate β-catenin, an event which further facilitates its nuclear translocation [34]. Immunofluorescence analysis showed that down-regulation of PTEN shifted β-catenin expression from the cytoplasmic membrane to the nucleus and downstream Nanog was also expressed in the nucleus. However, this phenomenon could be reversed by LY294002. These results suggested that the transcriptional activity of nuclear β-catenin depends on PTEN/PI3K/AKT axis activity. β-catenin up-regulates Nanog expression which may result in CSCs properties. This correlation is consistent with a previous report which demonstrated that β-catenin up-regulated Nanog expression in embryonic stem cells [19]. We aslo directly examined nuclear non-phosphorylated β-catenin in PTEN−/+ cells. Following the irradiation of PTEN− cells at 4 Gy, non-phosphorylated β-catenin was found to have translocated to the nucleus. Our data demonstrate that accumulation of nuclear β-catenin is associated with stemness and radioresistance in NPC. To the best of our knowledge, few studies have investigated the prognostic value of nuclear β-catenin activity in the radioresistance of NPC. The clinical significance of the current study is that these findings may provide potential biomarkers for predicting the sensitivity of NPC to radiotherapy.

In the present study, we found that decreased expression of PTEN and elevated expression of p-AKT, nuclear β-catenin and Nanog were significantly associated with poor prognosis in patients with NPC who had received radiotherapy. The expression of PTEN and Nanog is obviously correlated with nuclear, but not membranous or cytoplasmic β-catenin. We propose that the PTEN−/p-AKT+/nucleus β-catenin+/Nanog+ axis contributes to CSCs and radioresistance in patients with NPC.

In conclusion, our results show that PTEN plays a crucial role in the regulation of CSCs and radioresistance of NPC. PTEN− cells have CSCs properties that are resistant to radiation in NPC. PTEN exerts these effects through the downstream effectors PI3K/AKT/β-catenin/Nanog. Notably, nuclear β-catenin activation is essential for regulating the CSCs phenotype and radioresistance of NPC cells. Our results provide preclinical evidence to support the PTEN/PI3K/AKT/β-catenin/Nanog axis as a potential signaling pathway for CSCs and radioresistance of NPC.

## MATERIALS AND METHODS

### Cell culture and irradiation

All cell lines were purchased from the Shanghai Cell Biology Institute of the Chinese Academy of Sciences, China. The NPC cell lines CNE1, CNE2, SUNE1, 6-10B and 5-8F were cultured in DMEM medium (Hyclone, Logan, UT, USA) supplemented with 10% fetal bovine serum (FBS; Hyclone) and 100 U/ml penicillin/streptomycin (Gibco, Langley, OK, USA). The cells were cultured in humidified air with 5% CO_2_ at 37°C. The cells were then irradiated at the indicated doses using 250 kV orthovoltage X-rays and a linear accelerator (Elekta, Stockholm, Sweden).

### Fluorescence activated cell sorting

Cells were permeabilized using 0.1% Triton X-100 for 5 min on ice. Then cells were incubated with PTEN antibody (Abcam, Cambridge, MA, USA) at 1: 120 for 30 min at room temperature, and Alexa Fluor 488-conjugated secondary antibody (Abcam) was used at 1: 2000 at room temperature for 30 min. Finally, the cells were maintained at 4°C and the PTEN−/+ fraction was sorted using a FACSAria flow cytometer (BD Biosciences, San Jose, CA, USA). Data analysis was performed using FlowJo version 4.

### Clone formation assay

Cells treated with irradiation were collected and subsequently re-plated in a 30-mm culture dish at a density of 200–5000 cells/dish. Following culture for 14 days, the cells were fixed with 10% formalin and stained with 0.1% crystal violet; clones consisting of more than 50 cells were scored. The survival fraction was calculated by dividing the number of colonies formed by the number of cells plated. The average data were fitted into the single hit multitarget formula as follows: S=1-(1-e-D/D°)N. GraphPad Prism 5.0 software (GraphPad Software Inc., La Jolla, CA, USA) was used to draw the survival fraction curve.

### Tumor spheroid formation assay

Irradiated cells (1000 cells/ml) were cultivated in serum-free Ham's F-12 medium (Gibco), supplemented with B27 (1: 50; Gibco), 20 ng/ml Epidermal growth factor (EGF, Invitrogen, Grand Island, NY, USA) and 20 ng/ml Fibroblast growth factor (FGF, Invitrogen). The tumorspheres were counted under a microscope after culture for 72 h.

### Immunofluorescence

Cells were grown on the surface of cover slides and fixed with 4% paraformaldehyde. After rehydration in PBS, the fixed cells were incubated with primary antibodies: γ-H2AX, β-catenin, active β-catenin and Nanog (Abcam) at room temperature for 1 h or at 4°C overnight. Alexa594-conjugated goat anti-mouse IgG (Invitrogen) or Alexa488-conjugated goat anti-rabbit IgG (Invitrogen) were used as secondary antibodies. The nuclei were stained with DAPI. Sections were examined by confocal microscopy (Olympus-FV1000, Tokyo, Japan).

### Comet assay

Cells were digested and collected immediately, washed with PBS, and suspended in PBS. DNA damage was evaluated using the single-cell gel electrophoresis assay under alkaline conditions which was performed as described previously [35]. The comet slides were viewed with a fluorescence microscope and data were collected with a digital imaging system and analyzed using CASP software (Wroclaw, Poland).

### Western blot

Total protein was extracted from cells using cell lysis buffer. Proteins were loaded and separated on a 10% SDS-PAGE gel and then transferred to PVDF membranes. After blocking in 50 g/L non-fat milk in TBST (20 mmol/L Tris-HCl, 137 mmol/L NaCl, 1 g/L Tween 20, pH 7.6) for 2 h at room temperature, the membranes were incubated at 4°C overnight with the following primary antibodies: PTEN, AKT, p-AKT, β-catenin, active β-catenin, Nanog and GAPDH (Abcam). The membranes were then incubated for 1 h with HRP-conjugated secondary antibodies (Invitrogen, Logan, UT, USA). Finally, the membranes were visualized using the ECL-Plus detection system (Bio-Rad, Hercules, CA, USA).

### siRNA transfection and reagents

To knock down the expression of PTEN, we transfected small interfering RNA (siRNA) of PTEN or the negative control (NC) (Genepharma, Shanghai, China) into target cells using lipofectamine 2000 (Invitrogen, Carlsbad, CA, USA) according to the manufacturer’s protocol. LY294002, Perifosine and IGF-1 were purchased from Sigma-Aldrich (St. Louis, MO, USA).

### Lentiviral constructs and infection of NPC cells

Both the pLKO.1 lentiviral shRNA vector and control shRNA targeting GFP were purchased from Sigma-Aldrich. The β-catenin targeting sequence, GCTTGGAATGAGACTGCTGAT, was previously described [36]. The sense and antisense oligonucleotides were annealed and ligated into the pLKO.1 lentiviral vector. The viruses were then packaged in 293T cells. Viral production and infection of target cells were performed as previously described [37].

### Side population assay

Cells were suspended in DMEM/2% FBS at 1×10^6^ cells/ml. The cells were then dispersed into single cells, incubated with Hoechst 33342 dye (5 μg/ml; Sigma-Aldrich) either alone or in combination with verapamil (50 mmol/ml; Sigma-Aldrich) for 90 min at 37°C. Following incubation, the cells were washed with PBS and stained with propidium iodide (1 μg/ml; Sigma-Aldrich). Finally, the cells were maintained at 4°C for flow cytometric analysis using a FACSAria flow cytometer (BD Biosciences).

### qPCR

The survivin primer sequences were as follows: survivin forward 5' aagaactaccgcatcgccacc and survivin reverse 5′ agccagctccgccatt. Cells were harvested 24 h after irradiation. SYBR green quantitative PCR was performed using TaqMan assays (Applied Biosystems, Foster City, CA, USA) according to the manufacturer’s protocol.

### Immunohistochemistry

Nasopharyngeal carcinoma tissue microarrays were purchased from Shanghai Outdo Biotech. (Shanghai, China). These arrays included 70 cases of malignant tissues of the nasopharynx (various grades and stages). The last follow-up date was December 31st, 2015. All patients provided written informed consent. Paraffin-embedded sections were deparaffinized in xylene and rehydrated in a graded alcohol series. Antigen retrieval was performed by boiling the slide preparations in 10 mM sodium citrate buffer, pH 6.0. Staining was carried out as previously described [28] using anti-PTEN, p-AKT, β-catenin and Nanog antibodies (Abcam).

### Statistical analyses

A P-value < 0.05 was considered statistically significant. Data were analyzed with the SPSS 19.0 statistical software package and are shown as the means ± SD. Differences between groups were determined using the Student’s t-test or one-way analysis of variance (ANOVA). Graphs summarizing immunohistochemical staining results were analyzed using the chi-squared test. The Kaplan-Meier procedure was used to calculate survival probability estimates.
